# Molecular Targeting of the Oncoprotein PLK1 in Pediatric Acute Myeloid Leukemia: RO3280, a Novel PLK1 Inhibitor, Induces Apoptosis in Leukemia Cells

**DOI:** 10.3390/ijms16011266

**Published:** 2015-01-07

**Authors:** Na-Na Wang, Zhi-Heng Li, He Zhao, Yan-Fang Tao, Li-Xiao Xu, Jun Lu, Lan Cao, Xiao-Juan Du, Li-Chao Sun, Wen-Li Zhao, Pei-Fang Xiao, Fang Fang, Guang-Hao Su, Yan-Hong Li, Gang Li, Yi-Ping Li, Yun-Yun Xu, Hui-Ting Zhou, Yi Wu, Mei-Fang Jin, Lin Liu, Jian Ni, Jian Wang, Shao-Yan Hu, Xue-Ming Zhu, Xing Feng, Jian Pan

**Affiliations:** 1 Department of Hematology and Oncology, Children’s Hospital of Soochow University, Suzhou 215003, China; E-Mails: wangnn90s@163.com (N.-N.W.); szlizhiheng@hotmail.com (Z.-H.L.); zhaoheytr@163.com (H.Z.); taoyanfang1982@163.com (Y.-F.T.); xulixiao2013@hotmail.com (L.-X.X.); drlujun_sz@163.com (J.L.); cl2012@sohu.com (L.C.); wenlizhao69@163.com (W.-L.Z.); xiaopfdr@gmail.com (P.-F.X.); baseff77@gmail.com (F.F.); sky2skeleton@163.com (G.-H.S.); lyh072006@hotmail.com (Y.-H.L.); ligangmailbox@hotmail.com (G.L.); ray8302880@163.com (Y.-P.L.); rdxyy@163.com (Y.-Y.X.); huitingzhousdfey@163.com (H.-T.Z.); conan.005@stu.xjtu.edu.cn (Y.W.); sunnysnow12@163.com (M.-F.J.); flykf@163.com (L.L.); wj196312@vip.163.com (J.W.); hsy139@126.com (S.-Y.H.); xueming_zhu@aliyun.com (X.-M.Z.); 2 Department of Gastroenterology, the 5th Hospital of Chinese People’s Liberation Army (PLA), Yinchuan 750000, China; E-Mail: du_xiaojuan123@163.com; 3 Department of Cell and Molecular Biology, Cancer Institute (Hospital), Chinese Academy of Medical Sciences, Peking Union Medical College, Beijing 100021, China; E-Mail: sunlichao_1980@hotmail.com; 4 Translational Research Center, Second Hospital, The Second Clinical School, Nanjing Medical University, Nanjing 210000, China; E-Mail: ni_jian2008@163.com

**Keywords:** RO3280, pediatric acute myeloid leukemia (AML), polo-like kinase 1 (PLK1), apoptosis, oncogene target

## Abstract

Polo-like kinase 1 (PLK1) is highly expressed in many cancers and therefore a biomarker of transformation and potential target for the development of cancer-specific small molecule drugs. RO3280 was recently identified as a novel PLK1 inhibitor; however its therapeutic effects in leukemia treatment are still unknown. We found that the PLK1 protein was highly expressed in leukemia cell lines as well as 73.3% (11/15) of pediatric acute myeloid leukemia (AML) samples. PLK1 mRNA expression was significantly higher in AML samples compared with control samples (82.95 ± 110.28* vs.* 6.36 ± 6.35; *p* < 0.001). Kaplan-Meier survival analysis revealed that shorter survival time correlated with high tumor PLK1 expression (*p =* 0.002). The 50% inhibitory concentration (IC_50_) of RO3280 for acute leukemia cells was between 74 and 797 nM. The IC_50_ of RO3280 in primary acute lymphocytic leukemia (ALL) and AML cells was between 35.49 and 110.76 nM and 52.80 and 147.50 nM, respectively. RO3280 induced apoptosis and cell cycle disorder in leukemia cells. RO3280 treatment regulated several apoptosis-associated genes. The regulation of DCC, CDKN1A, BTK, and SOCS2 was verified by western blot. These results provide insights into the potential use of RO3280 for AML therapy; however, the underlying mechanisms remain to be determined.

## 1. Introduction

Acute leukemia is a family of serious medical conditions relating with an original diagnosis of leukemia. Acute leukemia is known as cancer of the blood which can cause death if not medicated [1]. Multiple small-molecule kinase inhibitors are currently being developed for leukemia treatment [2,3,4,5]. For example, selective FLT3 (fms-related tyrosine kinase 3) inhibitors, such as AC220 (quizartinib), have been proven clinically effective in acute myeloid leukemia (AML) patients with internal tandem duplications (ITD) in the gene of FLT3 [6,7]. Other inhibitors in development target mediators of downstream signaling pathways, such as mTOR [8] and MEK [9], or cell cycle machinery, such as aurora kinases [3,10] or cyclin-dependent kinases [11]. Recently, the polo-like kinase 1 (PLK1) inhibitor has showed promising effects in the treatment of acute leukemia. Polo-like kinases (PLKs) are a set of regulators that control mitotic progression [12]. PLK1, the best-characterized mammalian PLK, is a particularly attractive target for cancer drug development because most cancers require its activity [12,13,14,15].

PLK1 is a serine/threonine-protein kinase expressed during mitosis and overexpressed in multiple cancers, including acute leukemia [13]. PLK1 expression is increased in several types of cancers including urothelial carcinoma of bladder [16], renal cancer [17], breast cancer [18], prostate cancer [19], neuroblastoma [20], hepatocellular carcinoma [21], cervical carcinoma [22], and non-melanoma skin cancers [23]. PLK1 is also highly expressed in leukemic cell lines and over expressed in a majority of samples from patients with acute myeloid leukemia compared with normal progenitors [24]. And PLK1 knockdown by small interfering RNA also blocked proliferation of leukemic cell lines and the clonogenic potential of primary cells from patients [24]. PLK1 inhibition is a promising strategy for the treatment of AML. Furthermore, the expression of PLK1 in untransformed cells is much lower, which makes PLK1 a suitable target for the development of cancer-specific small molecule drugs. Supporting evidence of oncogenic properties of PLK1 comes from the overexpression studies of PLK1 in an NIH3T3 cell line [25,26]. These cells become capable of forming foci in soft agar due to PLK1 overexpression [27]. Depletion or inhibition of kinase activity of PLK1 is sufficient to induce cell-cycle arrest and apoptosis in cancer cell lines and in xenograft tumor models [28,29,30,31].

Inhibitors of PLK1 have been developed for potential human cancer therapy. NMS-P937 is a novel PLK1-specific inhibitor and shows high potency in proliferation inhibition on a large number of cell lines [28]. Another PLK1 inhibitor, BI2536, was tested against a panel of nine ALL cell lines at nanomolar concentrations. BI2536 strongly diminished colony formation capacity and increased apoptosis rates of these cells [32]. Co-treatment with BI2536 and vorinostat synergistically induced cell death in parental or imatinib mesylate-resistant BCR/ABL+ cells and primary CD34+ bone marrow cells but was minimally toxic to normal cells [33]. TAK-960 is another novel PLK1 inhibitor that has shown activity in several tumor cell lines, including those cells highly expressed multidrug-resistant protein 1 (MDR1) [29]. GW843682X is also a novel selective PLK1 inhibitor which caused accumulation of cells in the G_2_/M phase and mediated apoptosis of human leukemia cells [34].

Wovkulich and colleagues [30] have developed a novel PLK1 inhibitor, RO3280; RO3280 has potent inhibitory activity against PLK1, good selectivity against other kinases, and excellent* in vitro* cellular potency. However, the molecular function of this drug in leukemia is still unknown [30]. In the present study, RO3280 has been evaluated to further characterize its preclinical antitumor efficacy, and the molecular mechanism of action was explored with real-time PCR arrays.

## 2. Results and Discussion

### 2.1. Expression of PLK1 Is Upregulated in AML Cells and Pediatric AML Patients

**Figure 1:**
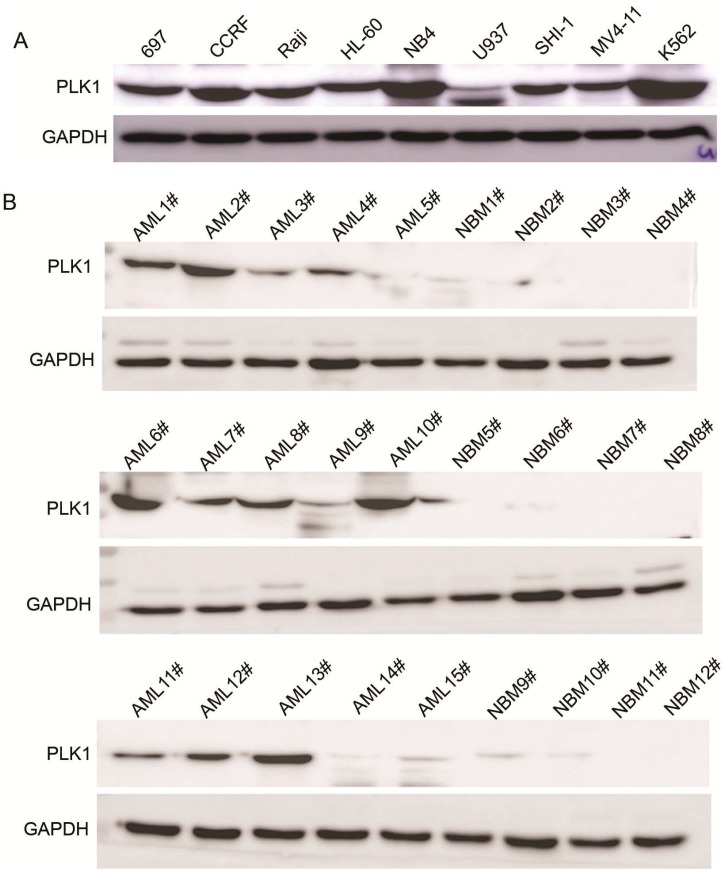

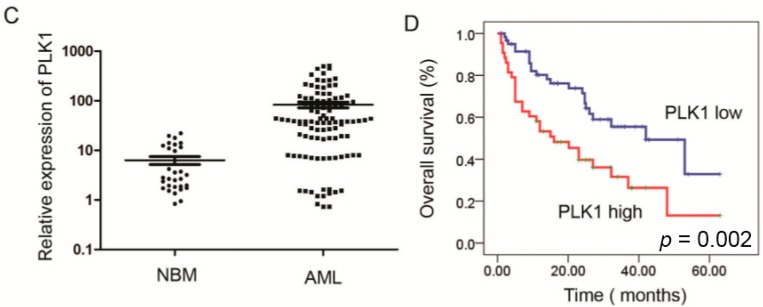
Expression of PLK1 is upregulated in AML cells and pediatric AML patients (**A**) Western blot analysis showing PLK1 protein expression in nine leukemia cell lines; (**B**) Western blot analysis showing PLK1 protein expression in 15 pediatric AML samples and 12 NBM samples; (**C**) Real-time PCR analysis of the PLK1 mRNA transcript levels in 105 pediatric AML samples and 30 NBM/ITP (normal bone marrow/idiopathic thrombocytopenic purpura) control samples; and (**D**) Kaplan-Meier survival analysis of 105 pediatric AML patients comparing high and low PLK1 expression (*p* = 0.002).

As reported previously, PLK1 is highly expressed in a broad set of cancer cell lines and overexpressed in a majority of cancer patient samples compared with normal progenitor cells. However, the expression of PLK1 in AML, and specifically pediatric AML, has not been clearly defined. We demonstrate that the expression of PLK1 is very high in AML cell lines, with the highest levels observed in CCRF, NB4, and K562 cells (Figure 1A). To examine the expression of PLK1 in pediatric AML samples, we obtained samples from 15 patients with pediatric AML and 12 control patients. High protein expression of PLK1 was observed in 73.3% (11/15) of the pediatric AML samples compared to 0% (0/12) of the normal bone marrow (NBM) control samples (Figure 1B). Real-time PCR was also used to examine the mRNA transcript levels of PLK1 in 105 pediatric AML samples and 30 NBM/ITP (idiopathic thrombocytopenic purpura) (control samples (Figure 1C)). PLK1 expression was significantly higher in the AML samples compared to the control samples (82.95 ± 110.28* vs.* 6.36 ± 6.35; *p* < 0.001). Bone marrow specimens were obtained from 105 pediatric patients with AML at the time of diagnosis, who presented at Children’s Hospital of Soochow University between 2000 and 2011. We suppose the high SD (standard deviation) values are related to the cDNA quality of samples. Examination of pediatric AML patient clinicopathology revealed that expression of PLK1 is related with FAB (French-American-Britain) and MRD (Minimal Residual Disease, Table 1). However, there were no significant differences in other clinical features such as sex, age, initial hemoglobin level, white blood cell counts, platelet counts, or chromosomal abnormalities between individuals with high and low PLK1 expression (Table 1). The prognostic significance of PLK1 expression was assessed in 105 Chinese pediatric AML patients with clinical follow-up records. Kaplan-Meier survival analysis revealed shorter survival times for patients with high PLK1 expression in tumors (*p =* 0.002, Table 2 and Figure 1C). Furthermore, multivariate analysis revealed that PLK1 expression is an independent prognostic factor in pediatric AML (*p* = 0.041, Table 3). In summary, our results demonstrate that PLK1 expression is heightened in patients with pediatric AML and in human myeloid leukemia cell lines. This indicates that PLK1 may be a suitable oncogene target for pediatric AML therapy.

**Table 1:** Association of polo-like kinase 1 (PLK1) expression with clinico-pathological characteristics in 105 pediatric acute myeloid leukemia (AML) samples.

Clinical Variables	No. of Patients	PLK1 Expression (*n*)		*p*
		Low	High	
Gender				
Male	42	27	15	0.373
Female	63	35	28	
Age (year)				
<6	60	35	25	0.864
≥6	45	27	18	
Leukocyte (/μL)				
>10,000	61	37	24	0.693
≤10,000	44	25	19	
FAB				
M1–M6	93	59	34	0.011
M7	12	3	9	
Cytogenetics				
Favorable	50	28	22	0.160
Intermediate	27	20	7	
Unfavorable	28	14	14	
MRD				
<0.25%	49	36	13	0.005
≥0.25%	56	26	30	

FAB: French-American-Britain; MRD: Minimal Residual Disease.

**Table 2:** Association of PLK1 expression with Kaplan-Meier survival in 105 pediatric AML samples.

Variable	No. of Patients	Over Survival	*p*
		Median ± SE	
Cytogenetics			
Favorable	50	46.664 ± 3.717	<0.001
Intermediate	27	29.220 ± 3.188	
Unfavorable	28	11.161 ± 1.827	
FAB			
M1–M6	93	36.113 ± 2.885	<0.001
M7	12	8.542 ± 1.820	
Leukocyte (/μL)			
>10,000	61	30.220 ± 2.974	0.803
≤10,000	44	33.631 ± 4.063	
MRD			
<0.25%	49	53.627 ± 3.151	<0.001
≥0.25%	56	18.893 ± 2.425	
PLK1 expression			
Low (<12.420)	62	39.319 ± 3.539	0.002
High (≥12.420)	43	24.054 ± 3.709	

**Table 3:** Cox multivariate analysis of PLK1 expression and clinico-pathological features in pediatric AML.

Variable	Odds Ratio	EXP (B) 95% CI	*p*
Cytogenetics			
Favorable *vs.* Intermediate and Unfavorable	6.164	2.477 (1.210–5.068)	0.013
MRD			
<0.25% *vs.* ≥0.25%	14.084	5.176 (2.193–12.214)	0.000
Leukocyte (/uL)			
>10,000* vs.* ≤10,000	0.200	1.138 (0.646–2.055)	0.655
FAB classification			
M7* vs.* M1–M6	7.148	2.683 (1.301–5.533)	0.008
PLK1 Expression			
Low* vs.* High	4.195	1.806 (1.026–3.179)	0.041

EXP (B) 95% CI: 95% CI (confidence intervals) of relative risk.

### 2.2. RO3280 Inhibits the Growth of Acute Leukemia Cells

**Figure 2:**
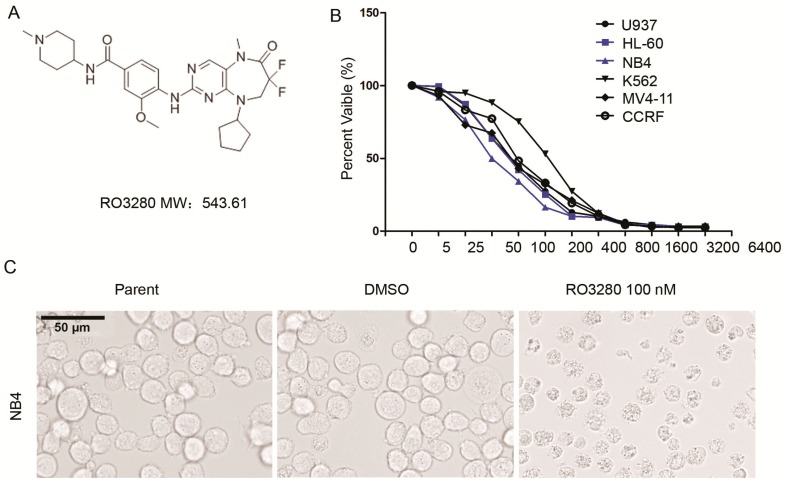

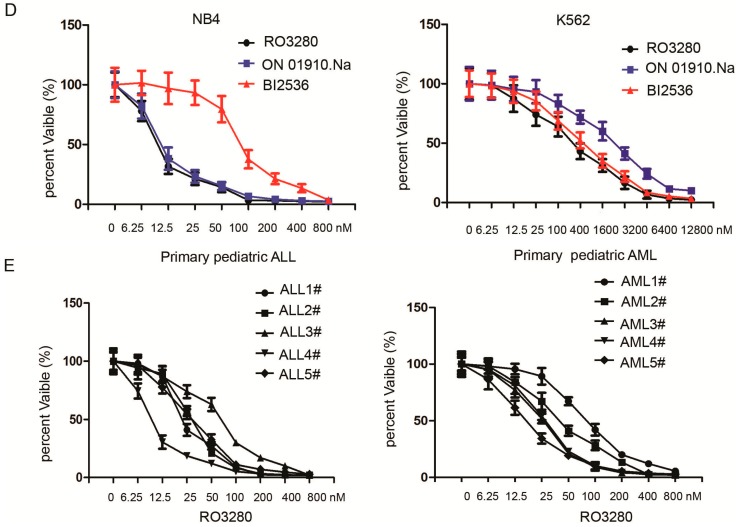
RO3280 inhibits the growth of acute leukemia cells. (**A**) Molecular structure of RO3280; (**B**) Viability and IC_50_ analysis of RO3280 in six leukemia cells. The following are the determined RO3280 IC_50_s: U937 186 nM, HL60 175 nM, NB4 74 nM, K562 797 nM, MV4-11 120 nM and CCRF 162 nM; (**C**) Micrographs were taken of NB4 cells treated with RO3280 100 nM or DMSO (scale bar = 50 μm); (**D**) The IC_50_ of three PLK1 inhibitors, RO3280, ON 01910.Na and BI2536, was analyzed in NB4 and K562 cells. IC_50_ in NB4 cells: RO3280 13.45 nM, ON 01910. Na 13.02 nM, and BI2536 87.65 nM. IC_50_ in K562 cells: RO3280 301 nM, ON 01910. Na 1606 nM, and BI2536 is 448 nM; and (**E**) The IC_50_ of RO3280 in primary acute lymphocytic leukemia (ALL) and AML cells was also analyzed: ALL 35.49–110.76 nM and AML 52.80–147.50 nM. Cell proliferation was calculated as a percentage of the DMSO treated control wells. The IC_50_ values were derived after plotting proliferation values on a logarithmic curve. Experiments were performed in quadruplicate and repeated twice.

The novel PLK1 inhibitor RO3280 decreased leukemia cell viability in a dose-dependent manner (Figure 2A,B). The RO3280 IC_50_ measurement was determined in several acute leukemia cell lines: U937 186 nM, HL60 175 nM, NB4 74 nM, K562 797 nM, MV4-11 120 nM, and CCRF 162 nM. RO3280 treatment could also dramatically impact cell morphology as observed in NB4 cells (Figure 2C).

In order to better understand the effective of RO3280, we compared it with other PLK1 inhibitors: Rigosertib (ON 01910. Na) and BI2536. The IC_50_ of these PLK1 inhibitors was analyzed in both NB4 and K562 cells (Figure 2D). In NB4 cells the following IC_50_ concentrations were determined: RO3280 13.45 nM, ON 01910. Na 13.02 nM, and BI2536 87.65 nM. In K562 cells the following IC_50_ concentrations were determined: RO3280 301 nM, ON 01910. Na 1606 nM, and BI2536 448 nM. To determine the effectiveness of RO3280 in primary leukemia, we determined the IC_50_ in ALL and AML cells. In primary ALL the IC_50_ of RO3280 is 35.49–110.76 nM (Figure 2E, Table 4) and in primary AML the IC_50_ of RO3280 is 52.80–147.50 nM (Figure 2E, Table 5). These results demonstrate that the PLK1 inhibitor RO3280 effectively inhibits the proliferation of leukemia cells.

### 2.3. RO3280 Induced Apoptosis and Cell Cycle Disorder in Leukemia Cells

**Figure 3:**
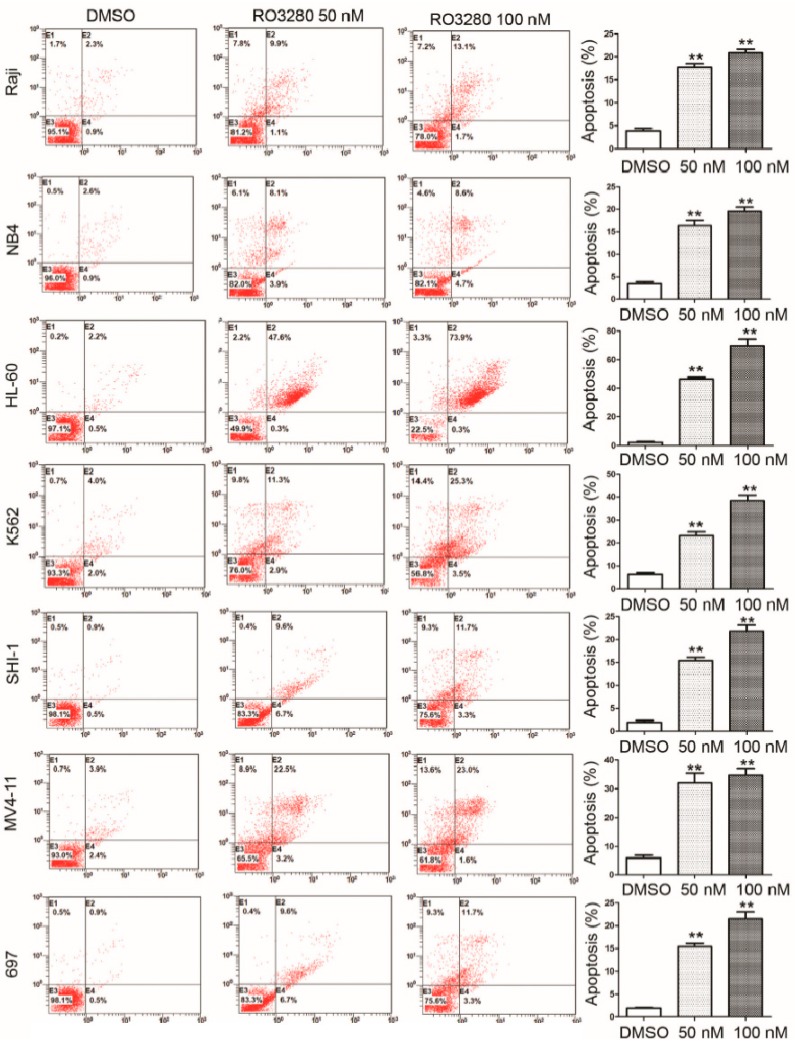

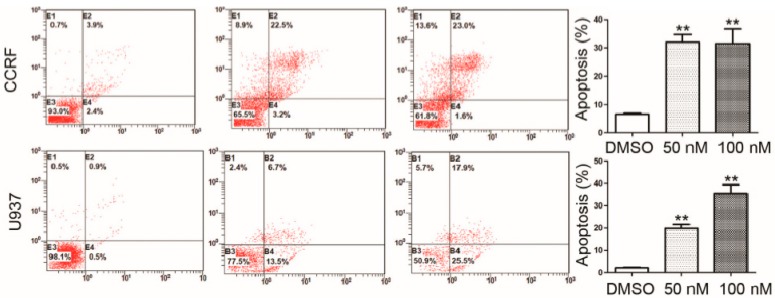
Annexin V analysis of apoptosis induced by RO3280 in acute leukemia cells. Annexin V staining of cells following a 24 h treatment with RO3280 at 50 or 100 nM compared with DMSO control mock treatment. All these analyses were repeated three times. ** *p* < 0.01.

**Figure 4:**
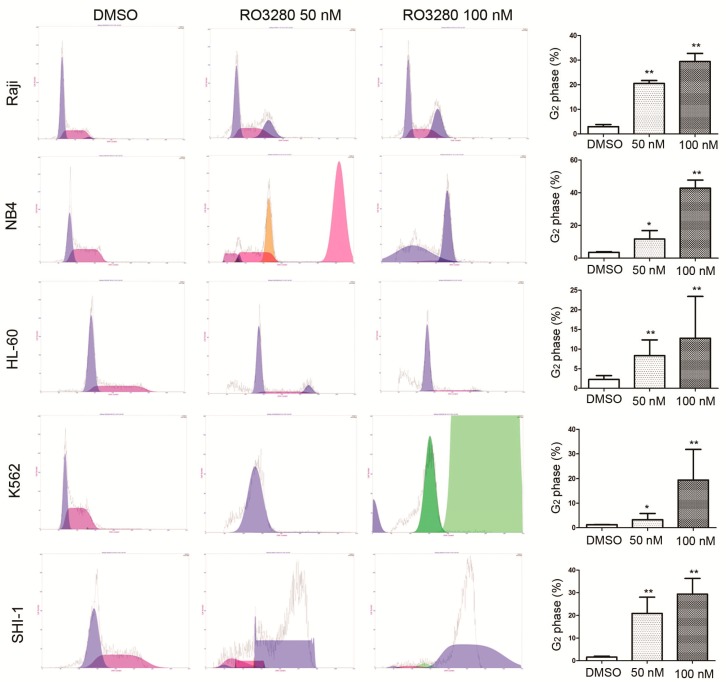

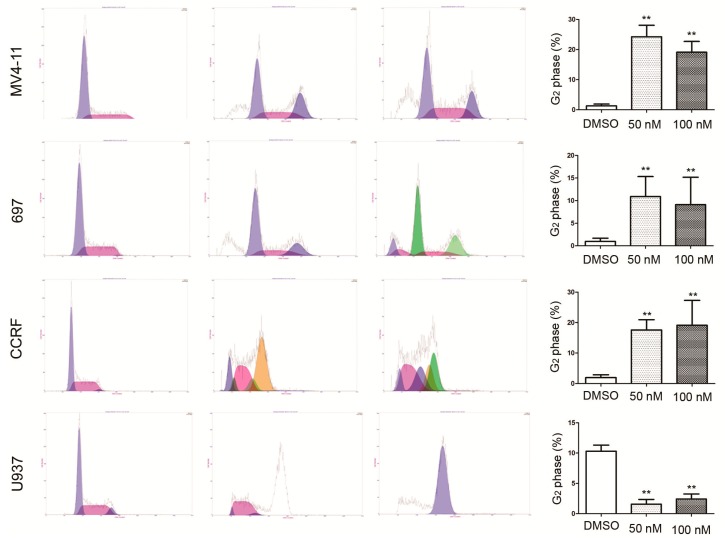
Cell cycle analysis of RO3280 induced cell cycle disorder in acute leukemia cells. Cell cycle analysis of nine leukemia cells treated for 24 h with RO3280 at 50 or 100 nM compared with DMSO control mock treatment. G_2_ phase of each group was analyzed and presented. All these analyses were repeated three times. * *p* < 0.05; ** *p* < 0.01. Left purple peak mean the G_1_ phase and right purple peak mean the G_2_ phase, red peak means the S phase; Pink, yellow and green peak mean the disorder of cell cycle.

**Figure 5:**
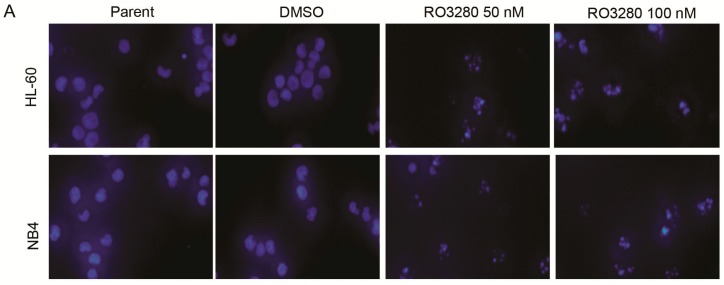

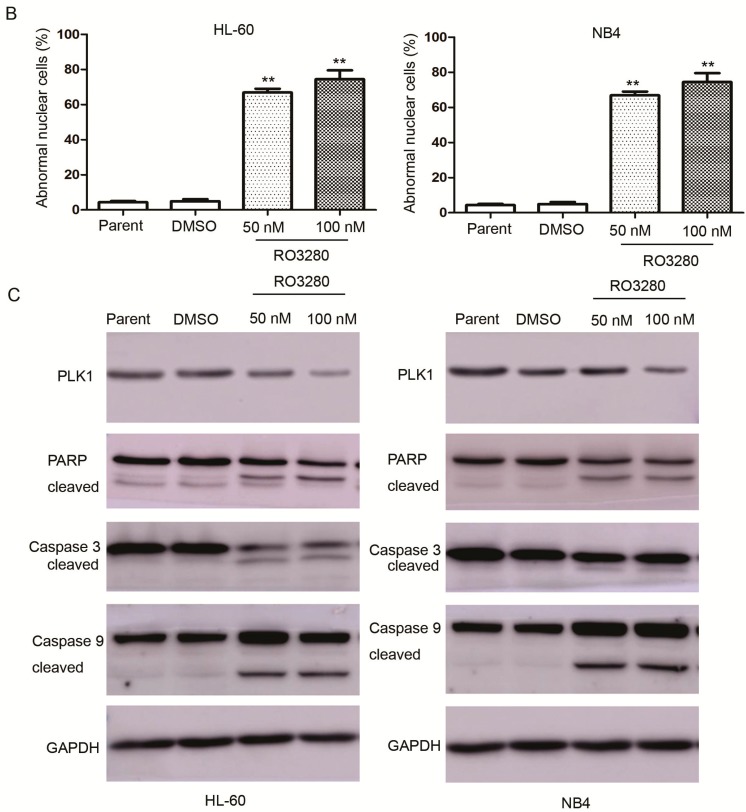
RO3280 induced DNA fragmentation and cleavage of apoptotic markers in acute leukemia cells. (**A**) Micrographs following Hoechst 33342 staining of cells treated with RO3280 (50 and 100 nM) for 24 h. This demonstrates the induction of DNA fragmentation and abnormal nuclear cell formation; (**B**) The abnormal nuclear cells were quantified and increased significantly with RO3280 treatment compared with mock treatment in both HL-60 and NB4 cells. ** *p* < 0.01; and (**C**) Western blot analysis of PARP, caspase-3, and caspase-9. After 24 h treatment with 50 or 100 nM RO3280, cleaved PARP and caspase-9 were detected in lysates from NB4 and HL-60 cells.

To determine if RO3280 induces apoptosis in leukemia cells, we assessed Annexin V staining, cell cycle disorder and caspase activation in leukemia cells after treatment. Cells treated with RO3280 for 24 h showed higher Annexin V staining compared with untreated cells (Figure 3). This indicates that RO3280 induces apoptosis in leukemia cells. Cell cycle disorder was determined by a cell cycle assay, which showed that RO3280 significantly induces cell cycle disorder in nine leukemia cell lines (Figure 4). Hoechst 33342 staining showed that 24 h RO3280 treatment induced DNA fragmentation and the formation of abnormal nuclear cells (Figure 5A). When either HL-60 or NB4 cells were treated with RO3280, there was a significant increase in abnormal nuclear cell formation compared with control DMSO treated cells (Figure 5B). Moreover, to clearly demonstrate that RO3280 causes apoptosis in leukemia cells, we assessed the following well-recognized markers of apoptosis: PARP, caspase-3, and caspase-9. After 24 h of treatment with RO3280 (50 or 100 nM), cleaved PARP and caspase-9 were observed in NB4 and HL-60 cells (Figure 5C). These results are consistent with the Annexin V staining and cell cycle analysis, demonstrating that RO3280 induces apoptosis in leukemia cells. This further suggests that RO3280 may have promising antitumor therapeutic applications.

**Table 4:** Pathologic features and inhibition of cell growth by RO3280 in primary culture cells of pediatric ALL.

Number	Gender	Age (Years)	Diagnosis	ALL Typing	Chromosome Analysis	Fusion Gene	Prednisone Sensitivity	PLK1	IC_50_ (nM)
1	Female	5	ALL	B	46, XY	Not detected	Sensitive	positive	35.39
2	Male	4	ALL	B	46, XY	*TEL*/*AML1*^+^	Sensitive	positive	30.79
3	Male	3	ALL	B	ALL/53–54, XY, +4, +6, +10, 12p+, +14, +17, +18, +20, +21	Not detected	Sensitive	positive	64.43
4	Female	4	ALL	B	46, XX	Not detected	Sensitive	positive	8.27
5	Female	4	ALL	B	ALL/53–55, XX, +X, 1q+, +4, +6, +10, +11, +15, +17, +21	Not detected	Sensitive	positive	36.01

**Table 5:** Pathologic features and inhibition of cell growth by RO3280 in primary culture cells of pediatric AML.

Number	Gender	Age (Years)	Diagnosis	AML Typing	Chromosome Analysis	Fusion Gene	PLK1	IC_50_ nM
1	Female	9	AML	M4	46, XX	*FLT3-ITD*	positive	88.65
2	Female	3	AML	M4	46, XX, inv (16) (p13;q22)	*CBF*/*MYH11*	positive	47.02
3	Male	4	AML	M5b	46, XY, −2,+10, t (10;10) (p13;q23)	*MLL*/*AF10*	positive	28.57
4	Male	12	AML	M2a	45, X, −Y, t (8;21) (q22;q22)	*AML*/*ETO*	positive	32.92
5	Female	1	AML	M4	46, XX, inv (16) (p13;q22)	Not detected	positive	19.20

### 2.4. Real-Time PCR Array Identifies Genes Implicated in the Effect of RO3280 Treatment

**Figure 6:**
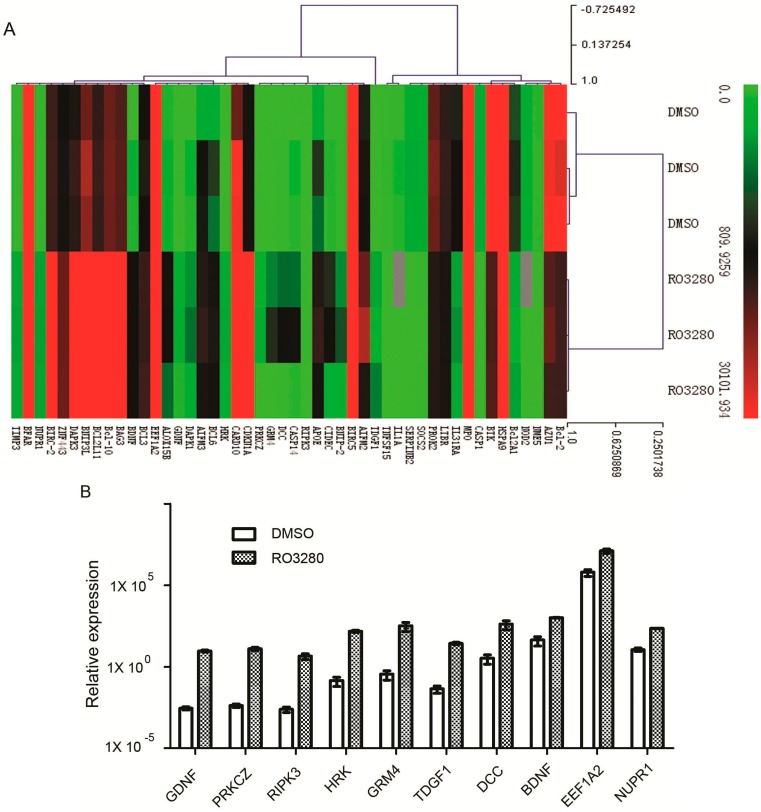

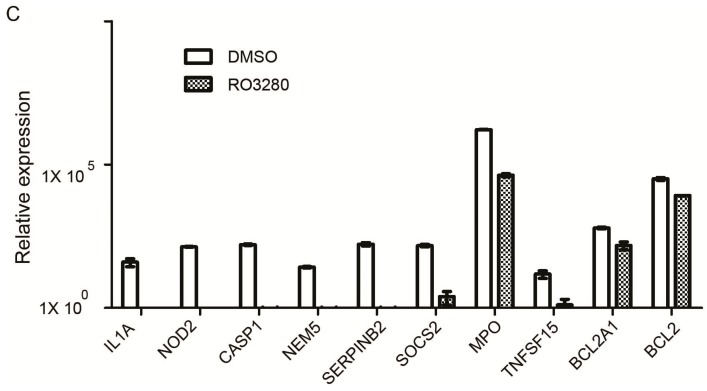
Real-time PCR array identifies genes implicated in the effect of RO3280 treatment. (**A**) Gene expression clustering of 370 key apoptosis genes in 50 nM RO3280-treated NB4 cells compared to DMSO-treated cells; (**B**) Relative expression of the most upregulated genes in RO3280-treated NB4 cells compared to DMSO-treated cells; and (**C**) Relative expression of the most downregulated genes in RO3280-treated NB4 cells compared to DMSO-treated cells.

**Figure 7:**
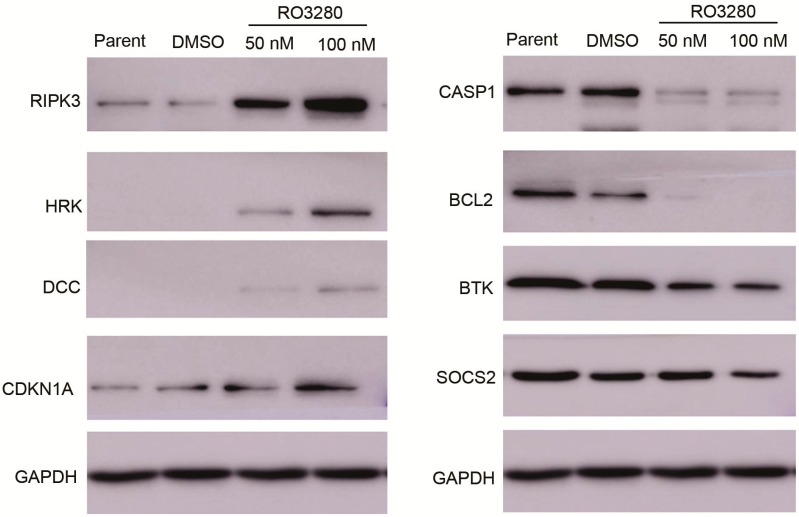
Western blot verification of real-time PCR array results. Western blot analysis of cells following a 24 h treatment with RO3280 at 50 or 100 nM compared with DMSO control mock treatment. The upregulation of DCC and CDKN1A and down regulation of BTK and SOCS2 in RO3280 treated cells. Protein lysates from treated cells were tested for expression levels by Western blot analysis.

In order to identify apoptosis and/or programmed cell death molecules implicated in the effects of RO3280, we used the SABioscience Human Apoptosis PCR Array PAHS-3012. With this real-time PCR array we analyzed and clustered the expression of 370 genes associated with apoptosis in DMSO or RO3280 treated cells (Figure 6A). The genes most significantly upregulated or downregulated are shown in Figure 6B,C, respectively. Examination of the array data revealed that 32 genes were significantly upregulated and 16 genes were significantly downregulated in RO3280 treatment group compared with DMSO control group (Table 6 and Table 7, respectively). The upregulated genes included protein kinase C zeta, receptor-interacting serine-threonine kinase 3, harakiri BCL2 interacting protein, DCC netrin 1 receptor, and cyclin-dependent kinase inhibitor 1A. The downregulated genes included interleukin 1α, nucleotide-binding oligomerization domain containing 2, caspase-1, apoptosis-related cysteine peptidase, serpin peptidase inhibitor clade B, B-cell CLL/lymphoma 2, and Bruton agammaglobulinemia tyrosine kinase. The RO3280-dependent upregulation of DCC and CDKN1A and downregulation of BTK and SOCS2 was verified by western blot analysis (Figure 7).

**Table 6:** Genes upregulated in NB4 cells treated with RO3280 compared with the DMSO control group.

Gene	Description	DMSO	RO3280	Fold Change	*p* Value
*GDNF*	glial cell derived neurotrophic factor	0.0029	9.4172	3262.8632	0.0162
*PRKCZ*	protein kinase C, zeta	0.0042	12.7679	3065.9848	0.0457
*RIPK3*	receptor-interacting serine-threonine kinase 3	0.0025	4.5249	1802.2433	0.0126
*HRK*	harakiri, BCL2 interacting protein	0.1471	150.6503	1023.9242	0.0246
*GRM4*	glutamate receptor, metabotropic 4	0.3625	335.0571	924.1889	0.0225
*TDGF1*	teratocarcinoma-derived growth factor 1	0.0451	27.3203	605.1673	0.0218
*DCC*	DCC netrin 1 receptor	3.4002	425.7855	125.2247	0.0225
*BDNF*	brain-derived neurotrophic factor	44.1739	1023.2023	23.1630	0.0007
*EEF1A2*	eukaryotic translation elongation factor 1 α 2	633,377.6354	12,905,456.2968	20.3756	0.0083
*NUPR1*	nuclear protein, transcriptional regulator, 1	11.4562	232.1343	20.2629	0.0001
*CIDEC*	cell death-inducing DFFA-like effector c	33.4482	667.6981	19.9621	0.0187
*CDKN1A*	cyclin-dependent kinase inhibitor 1A	2113.7362	36,484.2997	17.2606	0.0113
*BNIP-2*	BCL2/adenovirus E1B 19 kDa interacting protein 2	18.2415	306.7483	16.8160	0.0145
*BIRC5*	baculoviral IAP repeat containing 5	60,340.7297	981,719.5648	16.2696	0.0164
*TIMP3*	TIMP metallopeptidase inhibitor 3	15.1670	201.9090	13.3124	0.0031
*CASP14*	caspase 14, apoptosis-related cysteine peptidase	32.1570	410.5166	12.7660	0.0226
*CARD10*	caspase recruitment domain family, member 10	28,245.9365	329,683.7209	11.6719	0.0292
*ALOX15B*	arachidonate 15-lipoxygenase, type B	68.4589	796.1735	11.6299	0.0488
*APOE*	apolipoprotein E	411.8114	4632.2654	11.2485	0.0119
*BCL3*	B-cell CLL/lymphoma 3	950.2097	7763.8336	8.1707	0.0013
*BNIP3L*	BCL2/adenovirus E1B 19 kDa interacting protein 3-like	16,818.9831	130,854.4287	7.7802	0.0001
*DAPK1*	death-associated protein kinase 1	40.6424	287.7947	7.0811	0.0050
*AIFM2*	apoptosis-inducing factor, mitochondrion-associated, 2	1759.5718	12,454.0471	7.0779	0.0156
*BCL6*	B-cell CLL/lymphoma 6	385.7559	2574.1201	6.6729	0.0061
*AIFM3*	apoptosis-inducing factor, mitochondrion-associated, 3	764.9698	5057.6217	6.6115	0.0500
*ZNF443*	zinc finger protein 443	2001.5333	12,744.6420	6.3674	0.0009
*DAPK3*	death-associated protein kinase 3	5607.9528	30,206.6219	5.3864	0.0020
*BCL2L11*	BCL2-like 11 (apoptosis facilitator)	7094.6609	34,581.3650	4.8743	0.0000
*BIRC-2*	baculoviral IAP repeat containing 2	8366.5954	35,580.9121	4.2527	0.0007
*BAG3*	BCL2-associated athanogene 3	10,487.7923	34,964.8259	3.3339	0.0002
*Bcl-10*	B-cell CLL/lymphoma 10	13,170.6013	36,171.7865	2.7464	0.0000
*BFAR*	bifunctional apoptosis regulator	14,398.6434	31,271.8562	2.1719	0.0243

**Table 7:** Genes downregulated in NB4 cells treated with RO3280 compared with the DMSO control group.

Gene	Description	DMSO	RO3280	Fold Change	*p* Value
*IL1A*	interleukin 1α	39.8703	0.0000	0.0000	0.0316
*NOD2*	nucleotide-binding oligomerization domain containing 2	135.5059	0.0000	0.0000	0.0000
*CASP1*	caspase 1, apoptosis-related cysteine peptidase	161.0668	0.0011	0.0000	0.0001
*NME5*	NME/NM23 family member 5	26.4650	0.0006	0.0000	0.0000
*SERPINB2*	serpin peptidase inhibitor, clade B	168.2487	0.2504	0.0015	0.001
*SOCS2*	suppressor of cytokine signaling 2	150.3483	2.5012	0.0166	0.0006
*MPO*	myeloperoxidase	1,652,217.4204	42,599.1776	0.0258	0.0000
*TNFSF15*	tumor necrosis factor superfamily, member 15	15.2009	1.2803	0.0842	0.0360
*Bcl2A1*	BCL2-related protein A1	619.9195	152.6633	0.2463	0.0019
*Bcl-2*	B-cell CLL/lymphoma 2	31,688.9516	8210.5905	0.2591	0.0026
*BTK*	Bruton’s agammaglobulinemia tyrosine kinase	37,873.9816	9837.2022	0.2597	0.0001
*IL31RA*	interleukin 31 receptor A	823.6783	230.4415	0.2798	0.0009
*PROK2*	prokineticin 2	13,280.3733	4801.4889	0.3615	0.0004
*AZU1*	azurocidin 1	29,361.9342	10,755.3769	0.3663	0.0003
*LTBR*	lymphotoxin β receptor	6702.9536	2753.6837	0.4108	0.0004
*HSPA9*	heat shock 70 kDa protein 9	318,280.1754	141,258.3887	0.4438	0.0000

Serine/threonine-protein kinase PLK1, also known as polo-like kinase 1 (PLK-1) or serine/threonine-protein kinase 13 (STPK13). PLK1 is an early trigger for G2/M transition. PLK1 supports the functional maturation of the centrosome in late G_2_/early prophase and establishment of the bipolar spindle. PLK1 is an interesting molecule both as a biomarker and as a target for highly specific cancer therapy for several reasons. Firstly, it is over-expressed in many cancers and can serve as a biomarker. PLK1 is highly expressed in a broad range of tumors. Secondly, the expression of PLK1 in untransformed cells is not nearly as high, which makes it a discriminating target for the development of cancer-specific small molecule drugs. Medical experts are now paying more attention to the role of PLK1 in acute myeloid leukemia [35]. Here we show that PLK1 is highly expressed in all AML cell lines tested and in 73.3% (11/15) of the pediatric AML samples compared to 0% (0/12) of the NBM control sample. Real-time PCR analysis of 105 pediatric AML samples demonstrated significantly upregulated PLK1 expression in the AML samples compared to the control samples. Sometimes the results of the RT-PCR were not very accurate when the cDNA quality of samples was not very high. Thus, it is best to utilize Western blot analysis in future studies. Kaplan-Meier survival analysis of 105 pediatric AML patients revealed shorter survival time correlated with high PLK1 expression in tumors. Furthermore, multivariate analysis revealed that PLK1 expression is an independent prognostic factor in pediatric AML. In summary, our results are indicate for the first time that PLK1 may be a good oncogene target in samples from pediatric AML patients and human myeloid leukemia cell lines.

Several PLK1 inhibitors have been discovered and show significant anticancer effects. ATP-competitive inhibitor BI 6727 [36,37] is currently in clinical trials for cancer treatment. PLK1 inhibition with BI2536 leads to apoptotic effects in SCCHN cell lines [38]. BI2536 is a potent PLK1 inhibitor with IC_50_ of 0.83 nM. BI2536 also shows 4- and 11-fold greater selectivity against PLK2 and PLK3, and its effects in leukemia have been reported by others [32]. TAK-960 is currently undergoing Phase I evaluation in adult patients with advanced solid malignancies [39]. ON 01910. Na inhibits mitotic progression and induces apoptosis in most cancer cell lines [40], is currently in a phase I study for treating adult patients with advanced solid malignancies [41]. Rigosertib (ON-01910) is a PLK1 inhibitor with IC_50_ of 9 nM. As presented here, in leukemia cells, RO3280 is more effective than Rigosertib (ON-01910). Wovkulich and colleagues recently developed RO3280, a novel PLK1 inhibitor [30]. RO3280 is a potent, highly selective inhibitor of PLK1 with IC_50_ of 3 nM. Until now, we know that PLK1 is a target of RO3280 and RO3280 has nearly no effect on PLK2 and PLK3. However, the effectiveness of RO3280 in leukemia treatment remains an open question. In this report we demonstrate that RO3280 inhibits leukemia cell proliferation in a dose-dependent manner and causes apoptosis. This suggests that RO3280 may be a promising antitumor treatment for leukemia. Indeed the potential of PLK1 inhibitors in treatment is promising, and combinations of PLK1 inhibitors with other anticancer drugs might offer a larger range of opportunities for cancer therapy [42].

Real-time PCR Array System is ideal tool for analyzing the expression of a focused panel of genes [43]. In this study, we used real-time PCR arrays to analyze the effect of RO3280 treatment on apoptotic gene regulation. Compared to an untreated control group, 32 genes related to apoptosis were significantly upregulated and 16 genes were significantly downregulated after RO3280 treatment. This confirmed previous reports that CDKN1A, CASP1, and BCL2 are regulated by PLK1 inhibition. PLK1 is a checkpoint protein whose role spans all of mitosis and includes DNA repair. PLK1 has been reported as a negative regulator of CDKN1A (p21) [44]. When cancer cells are treated with PLK1 inhibitors, p21 is increased in the cytoplasm. By contrast, deficiency of p21 renders tumor cells more susceptible to Polo-like kinase 1 inhibition [45]. A rrecent study showed that PLK1 inhibition causes post-mitotic DNA damage and senescence in a range of human tumor cell lines [46]. Small-molecule PLK1 inhibitors and PLK1 genetic knock-down resulted in the induction of DNA double-strand breaks and senescence in a range of human tumor cell lines. Molecular function of PLK1 is very complicated and in metastatic alveolar rhabdomyosarcoma (aRMS), PLK1 interacted with and phosphorylated PAX3-FOXO1 leading to protein stabilization. PLK1 inhibition led to elevated ubiquitination and rapid proteasomal degradation of this PAX3-FOXO1 chimeric oncoprotein [47]. Our study also identified some novel genes such as RIPK3, HRK, DCC, and BTK regulated by PLK1 inhibition. While each of these genes has a function related to apoptosis, many are additionally involved in separate cellular processes. RIPK3 is a component of the tumor necrosis factor (TNF) receptor-I signaling complex and a key regulator of TNF-induced necrosis [48]. The DCC gene encodes a netrin 1 receptor that partially localizes to lipid rafts and induces apoptosis in the absence of ligand. The receptor functions as a tumor suppressor and is frequently mutated or down regulated in colorectal [49] and ovarian cancers [50]. HRK promotes apoptosis in mammalian cells by interacting with the apoptotic inhibitors BCL-2 and BCL-X_L_ via its BH3 domain [51]. Recent research indicates that PLK1 is an important regulator of PTEN during cell cycle progression [52]. Our research now indicates that PLK1 may also regulate RIPK3, DCC, and HRK expression. Until now, the regulation mechanism of PLK1 is still unclear. Thus, our results may provide new clues into the molecular mechanism of apoptosis induced by RO3280 in leukemia cells.

## 3. Experimental Section

### 3.1. Cell and Culture Conditions

Leukemia cell lines HL-60, MV4-11, U937, DAMI and K562 were obtained from the American Type Culture Collection (ATCC, Manassas, VA, USA). CCRF, Raji, Jurkat, 697 and SHI-1 cell lines (gifts from Wang Jian-Rong, The Cyrus Tang Hematology center of Soochow University, Suzhou, China). All cell lines were maintained at 37 °C in RPMI 1640 (Gibco, Carlsbad, CA, USA) supplemented with 10% fetal bovine serum (Invitrogen, Carlsbad, CA, USA). RO3280 (Cat: S7248; Selleck Chemicals, West Paterson, NJ, USA) was dissolved in DMSO (Cat: D4540; Sigma-Aldrich, St. Louis, MO, USA).

### 3.2. Patients and Samples

Bone marrow specimens were obtained from 105 pediatric patients with AML at the time of diagnosis, who presented at Children’s Hospital of Soochow University between 2000 and 2011. Ethical approval was provided by the Children’s Hospital of Soochow University Ethics Committee (Nos. SUEC2000-021 and SUEC2011-037), and written informed consent was obtained from the parents or guardians. AML diagnosis was made in accordance with the revised French-American-British (FAB) classification. Patients’ clinical and laboratory features are summarized in Table 1. Additionally, 23 healthy donors’ bone marrow samples and seven patients with ITP were analyzed as controls. Bone marrow mononuclear cells (BMNCs) from patients were isolated within 2 h using Ficoll solution when bone marrow samples were harvested.

### 3.3. CD34+ Cell Purification

For CD34+ cell selection, the Miltenyi immunoaffinity device (VarioMACS 130-046-703) was used according to the manufacturer’s instructions (Miltenyi Biotech, Auburn, CA, USA). Briefly, the CD34+ cells are magnetically labeled with CD34 MicroBeads. Then, the cell suspension is loaded onto a MACSR column which is placed in the magnetic field of a MACS Separator. The magnetically labeled CD34+ cells are retained within the column. The unlabeled cells run through; CD34+ cells were adsorbed on the magnetic poles. After removing the column from the magnetic field, the magnetically retained CD34+ cells can be eluted as the positively selected cell fraction.

### 3.4. Quantitative Reverse-Transcription PCR for PLK1

Quantitative real-time PCR was performed to determine the expression levels of *PLK1* genes. Total RNA was reverse transcribed using the Reverse Transcription Kit, according to the manufacturer’s protocol (Applied Biosystems Inc., Foster City, CA, USA). The real time PCR primers used to quantify GAPDH expression were: Forward (F): 5'-AGAAGGCTGGGGCTCATTTG-3' and Reverse (R): 5'-AGGGGCCATCCACAGTCTTC-3' and for PLK1 were: F: 5'-AGTCGACCACCTCACCTGTC-3' and R: 5'-GCCCCTCACAGTCCTCAATA-3'. Expression of PLK1 was normalized to endogenous GAPDH expression. Real-time PCR array analysis was according to the MIQE Guidelines [53] and performed in a total volume of 20 µL including 1 µL of cDNA, primers (0.2 mM each) and 10 µL of SYBR Green mix (Roche, Basel, Switzerland). Reactions were run on a Lightcycler 480 (Roche) using universal thermal cycling parameters (95 °C for 5 min, 45 cycles of 10 s at 95 °C, 20 s at 60 °C and 15 s at 72 °C; followed by a melting curve: 10 s at 95 °C, 60 s at 60 °C and continued melting). The results were obtained using the sequence detection software of the Lightcycler 480 and analyzed using Microsoft Excel. For quality control purposes, melting curves were acquired for all samples. The comparative *C*_t_ method was used to quantify gene expression. The target gene expression level was normalized to expression of the housekeeping gene glyceraldehyde 3-phosphate dehydrogenase (GAPDH) within the same sample (−Δ*C*_t_), and then the relative expression of each gene was calculated using Log 2 (−Δ*C*_t_).

### 3.5. Cell Proliferation and Viability Assay

Leukemia cells or primary leukemia cells (2 × 10^4^) were seeded in 96-well plates overnight and incubated with DMSO, or increasing concentrations of RO3280 (0.05–120 μM) for 24 h. The same volume of DMSO added to the vehicle treated wells. Each drug concentration was replicated four times. Then, 10 μL CCK8 (Dojindo Molecular Technologies, Tokyo, Japan) solution was added to each well, incubated at 37 °C for 2–4 h and the optical density (OD) values were measured at 450 nm using a scanning multi-well spectrophotometer (BioRad Model 550, Hercules, CA, USA). Relative survival rate was calculated from the absorbance values compared with the control group. The proliferation of cells was calculated as a percentage of the DMSO-treated control wells with 50% inhibitory concentration (IC_50_) values derived after plotting proliferation values on a logarithmic curve. The IC_50_ of PLK1 inhibitor was calculated by Graph Prism software (GraphPad-Prism Software Inc., San Diego, CA, USA).

### 3.6. Cell Cycle Analysis

Firstly, leukemia cells were collected and washed with PBS for 5 min by centrifugation at 125× *g*. Then, cells were fixed with paraformaldehyde and transparented with 0.5% Triton X-100 for 10 min. After that, cells were resuspended and incubated for 30 min at 37 °C in staining solution containing 1.5 μmol/L propidium iodide (P4170; Sigma-Aldrich, St. Louis, MO, USA) and 25 μg/mL RNase A The samples (1 × 10^4^ cells) were analyzed with a Beckman Gallios™ Flow Cytometer (Beckman, Krefeld, Germany).

### 3.7. Apoptosis Assay

Apoptosis assay was according to the manual operation of BD Annexin V Staining Kit (Cat: 556420; BD Biosciences, Franklin Lakes, NJ, USA). Briefly, cells were washed two times with cold PBS and then cells were resuspended in 1× Binding Buffer at a concentration of ~1 × 10^6^ cells/mL. Then 100 µL of the solution (~1 × 10^5^ cells) was transferred to a 5 mL culture tube. Annexin V and PI (Propidium Iodide) 5 µL/test was added. Cells were gently mixed and incubated for 15 min at RT in the dark. 400 µL of 1× Binding Buffer was added to each tube and the cells were analyzed by flow cytometry within one hour.

### 3.8. Western Blot Analysis

Cellular proteins were extracted in 40 mM Tris-HCl (pH 7.4) containing 150 mM NaCl and 1% (*v*/*v*) Triton X-100, supplemented with protease inhibitors for western blot analysis. Equal amounts of protein were resolved on 12% SDS-PAGE gels, and then transferred to a PVDF membrane (Millipore, Bedford, MA, USA). Blots were blocked and probed with antibodies against Caspase 3 (Cat: 9661S, 1:1000; Cell Signaling Technology Inc., Danvers, MA, USA), Caspase 9 (Cat: 4501S 1:1000, Cell Signaling Technology Inc.), PARP (Cat: 9542S, 1:1000; Cell Signaling Technology Inc.), PLK1 (Cat: 4535S, 1:1000; Cell Signaling Technology Inc.), RIPK3 (Cat: 13526S,1:1000; Cell Signaling Technology Inc.), HRK (Cat: ab45419, 1:1000; Abcam Trading (Shanghai) Company Ltd., Shanghai, China), DCC (Cat: 3858S, 1:1000; Cell Signaling Technology Inc.), CDKN1A(Cat: 2947S, 1:1000; Cell Signaling Technology Inc.), CASP1 (Cat: 2225S, 1:1000; Cell Signaling Technology Inc.), BCL2 (Cat: ab7973, 1:1000; Abcam Trading (Shanghai) Company Ltd.), BTK(Cat: 3533S, 1:1000; Cell Signaling Technology Inc.), SOCS2 (Cat: 2779S, 1:1000; Cell Signaling Technology Inc.), GAPDH (1:5000; Sigma). After washing three times, the blots were incubated with horseradish peroxidase-conjugated secondary antibodies for 1 h and visualized using an enhanced chemiluminescence kit (Pierce, Rockford, IL, USA). Finally, the protein bands were visualized after exposure of the membrane to Kodak X-ray film (Kodak, Rochester, NY, USA).

### 3.9. Hoechst 33342 Staining Analysis

Cells were seeded into 6-well plates, and then treated with RO3280 (50 or 100 nM) and cultured at 37 °C for 24 h, stained with 0.1 µg/mL Hoechst 33342 (Sigma) for 5 min, then observed with filters for blue fluorescence under fluorescence microscopy (OLYMPUS IX71; Olympus Corporation, Tokyo, Japan). Abnormal nuclear cells were counted between the RO3280 treatment group and DMSO control group.

### 3.10. Real-Time PCR Array Analysis

Samples from each group were submerged in 2 mL Trizol (Invitrogen) for RNA extraction, and stored at −80 °C until further processed. cDNA synthesis was performed on 4 μg of RNA in a 10 μL sample volume using SuperScript II reverse transcriptase (Invitrogen) as recommended by the manufacturer. Real-time PCR array (SABioscience Human Apoptosis PCR Array PAHS-3012, Frederick, MD, USA) analysis was performed in a total volume of 20 μL including 2 μL of cDNA, primers (0.2 mM each) and 10 μL of SYBR Green mix (Roche). For gene expression quantification a comparative *C*_t_ method was used. Gene expression levels for each sample were normalized to the expression level of the housekeeping gene encoding Glyceraldehyde 3-phosphate dehydrogenase (GAPDH) within a given sample (−Δ*C*_t_); the relative expression of each gene was calculated with 10^6^ × Log 2 (−Δ*C*_t_). Statistical significance of gene expression was calculated with the *t*-test using SPSS 11.5 software (Chicago, IL, USA).

### 3.11. Statistical Analysis

Each experimental condition was performed three times, and these replicates were presented in results. All values are presented as means ± SEM. Student’s paired *t*-test was applied to reveal statistical significances*. p* values less than 0.05 were considered significant. Statistical analyses were performed using SPSS Software for Windows.

## 4. Conclusions

In this study, we identified PLK1 as a possible oncoprotein target in pediatric AML patient samples and human myeloid leukemia cell lines. Kaplan-Meier survival analysis revealed shorter survival time correlated with high PLK1 expression in tumors. Our findings also showed for the first time that RO3280 treatment inhibits cell proliferation and induces apoptosis in leukemia cells. Real time PCR array analysis demonstrated that 32 genes are significantly upregulated and 16 genes are significantly downregulated after RO3280 treatment. This is the first indication of *RIPK3*, *HRK*, *DCC*, and *BTK* gene regulation reported with RO3280 treatment. These results may provide new insights into the molecular mechanism of RO3280-induced apoptosis; however, further research will be required to determine the underlying details. Taken together, our findings suggest that RO3280 may be a suitable drug candidate for the treatment of pediatric AML.
